# The Spherical Evolutionary Multi-Objective (SEMO) Algorithm for Identifying Disease Multi-Locus SNP Interactions

**DOI:** 10.3390/genes15010011

**Published:** 2023-12-20

**Authors:** Fuxiang Ren, Shiyin Li, Zihao Wen, Yidi Liu, Deyu Tang

**Affiliations:** 1 College of Medical Information Engineering, Guangdong Pharmaceutical University, Guangzhou 510006, China; renfuxiang163@163.com (F.R.); shiyyy0326@163.com (S.L.); 17803873294@163.com (Y.L.); 2 College of Mathematics and Informatics, College of Software Engineering, South China Agricultural University, Guangzhou 510642, China; 3 Faculty of Information Technology, Monash University, Melbourne, VIC 3800, Australia

**Keywords:** disease models, single-nucleotide polymorphisms, biogenetic markers, multi-locus SNP interactions, spherical evolutionary algorithms, multi-objective optimization

## Abstract

Single-nucleotide polymorphisms (SNPs), as disease-related biogenetic markers, are crucial in elucidating complex disease susceptibility and pathogenesis. Due to computational inefficiency, it is difficult to identify high-dimensional SNP interactions efficiently using combinatorial search methods, so the spherical evolutionary multi-objective (SEMO) algorithm for detecting multi-locus SNP interactions was proposed. The algorithm uses a spherical search factor and a feedback mechanism of excellent individual history memory to enhance the balance between search and acquisition. Moreover, a multi-objective fitness function based on the decomposition idea was used to evaluate the associations by combining two functions, K2-Score and LR-Score, as an objective function for the algorithm’s evolutionary iterations. The performance evaluation of SEMO was compared with six state-of-the-art algorithms on a simulated dataset. The results showed that SEMO outperforms the comparative methods by detecting SNP interactions quickly and accurately with a shorter average run time. The SEMO algorithm was applied to the Wellcome Trust Case Control Consortium (WTCCC) breast cancer dataset and detected two- and three-point SNP interactions that were significantly associated with breast cancer, confirming the effectiveness of the algorithm. New combinations of SNPs associated with breast cancer were also identified, which will provide a new way to detect SNP interactions quickly and accurately.

## 1. Introduction

The rapid development of high-throughput genotyping and sequencing technologies has led to the detection of a large amount of genetic data in the genome. Among them, single-nucleotide polymorphisms (SNPs) are the most common and abundant form of genetic variation, which refers to polymorphisms in DNA sequences that occur as a result of a single deoxyribonucleotide variant in a specific location in the genome [1]. The DNA sequences of individuals contain more than 3 million SNPs, of which approximately 93% of genes contain at least one SNP [2,3,4]. These large amounts of SNP genetic data contain dense information, and how to efficiently mine disease-causing SNP interactions from genome-wide data is the key to solving combinatorial explosion.

In the early days, genome-wide association studies (GWAS) focused on single genotype–phenotype associations [5]. However, due to the complex regulatory mechanisms in the human genome, multiple genetic variants can combine to interact with each other, leading to the emergence of a specific phenotype that may manifest as a complex disease (Alzheimer’s disease, breast cancer, schizophrenia) [6,7,8]. These interactions between multiple genetic variants when co-expressing a specific phenotype are called multi-locus SNPs or epistatic interactions [9,10]. Multi-locus SNP interactions can reveal the largely unexplained heritability of complex diseases and are essential for understanding the relationship between genotype and phenotype, for understanding disease susceptibility, and for treating genetic diseases [11].

According to the optimization strategy, existing SNP interaction detection methods can be broadly classified into four categories: exhaustive search, random search, depth-first, and intelligent algorithms. Among these, the most direct and simplest approach for detecting SNP interactions is the exhaustive search algorithm. The Multifactor Dimensionality Reduction (MDR) algorithm, as proposed by Ritchie and colleagues [12], serves as an exemplary representative of an exhaustive search, primarily centering on the stratification of genotypes into low-risk and high-risk groups to curtail the search space. The BMDR algorithm [13] augments the accuracy of predictive error rate estimation for small sample sizes. Nonetheless, an exhaustive search necessitates substantial computational resources, and as the order increases, it exhibits exponential growth, thus consuming an inordinate amount of time.

The stochastic search algorithm operates through random sampling to detect SNP interactions. The SNPHarvester [10] algorithm, as expounded in [10], undertakes different local search iterations by probing various combinations within the composite space. A stochastic search significantly diminishes the search domain and expedites the detection of SNP interactions. Nevertheless, the performance of a stochastic search hinges on the quantity of its sampling, and the substantial number of samples coupled with high-dimensional features epitomizes the attributes of SNP big data, thereby engendering challenges in data processing.

A depth-first search perseveres in uninterrupted succession until a certain quantity of combinations is achieved or until no further meaningful combinations can be discerned [14]. Notably, the Fast Depth-First Heuristic Search with Interaction Weights (FDHE-IW) [15] algorithm is founded upon the interaction weight. It incrementally constructs SNP combinations, enabling the swift detection of high-order SNP interactions. Furthermore, the ELSSI algorithm, an amalgamation of various detection mechanisms [16], assesses each subset of SNP combinations individually via a single detector, thus assigning scores accordingly.

Intelligent algorithms are conceived to emulate the survival-of-the-fittest principle found in the natural world, yielding remarkable effectiveness in addressing various optimization challenges. They epitomize a heuristic search strategy, guided by heuristics to govern high-level interactions, exemplified by the EpiACO algorithm [17] and the NHSA-DHSC algorithm [18].The EACO [19] algorithm embraces a multi-threshold spatially equitable alleviation as its heuristic selection, assessing associations by computing the ratio of mutual information to the Gini index and pinpointing significant combinations through inflection points on the metric of association. The MP-HS-DHSI [20] algorithm comprises three phases: exploration of candidate solutions, validation via the G-test, and resolution via MDR. The Interaction Pattern Pursuit (IPP) [4] algorithm leverages differential privacy (DP) to craft a judicious high-level privacy preservation strategy through perturbation of multi-objective functions. Owing to its positive feedback and a more confined search space, the heuristic search has outshone exhaustive and random search algorithms, evolving into a popular search strategy for detecting SNP interactions [3]. Nonetheless, it is susceptible to local optima, potentially forfeiting the global optimum. Therefore, the development of novel and effective methods for detecting SNP interactions is an imperative future task.

The detection of disease-related biogenetic marker SNP interactions faces severe computational challenges, and although many detection methods have been proposed, the current methods still suffer from the problems of slow computation and the possibility to easily fall into local optimality. In order to reduce the computational burden and mine the optimal combinations of disease-causing SNP interactions as quickly and accurately as possible, this paper proposes a spherical evolutionary multi-objective (SEMO) algorithm. The algorithm proposes a spherical evolutionary mechanism with memory, which adaptively records the values of search factors in the current generation according to the fitness values of the current group winners and uses the parameter adaptive mechanism to store the historical memory set. Meanwhile, a multi-objective fitness function based on the idea of decomposition is adopted and combined with two approximate normalization methods, using K2-Score and LR-Score statistical mathematical models as the objective function of the algorithm’s evolutionary iteration. By automatically storing a record of optimal solutions, SEMO is able to maintain the diversity as well as effectiveness of the solutions and improve the quality of the solutions accordingly. To evaluate the detection capability of the method, we conducted experiments on a simulated dataset and compared the performance with that of EACO [19], EpiACO [17], FDHEIW [15], MP-HS-DHSI [20], NHSA-DHSC [18], and SNPHarvester [10]. The results show that SEMO has advantages over all other methods. In addition, the practical feasibility of SEMO was experimentally validated using the real disease dataset (WTCCC).

## 2. Materials and Methods

Based on the definition of SNP correlation, multi-locus SNP interactions associated with disease can be transformed into a heuristic combinatorial optimization problem. It can be mathematically described as finding the optimal SNP combination to predict the phenotype as accurately as possible, where the SNPs in each combination have nonlinear interactions on the phenotype. During the search computation of the spherical evolutionary multi-objective algorithm proposed in this paper, a parameter adaptive mechanism was used, preserving the well-performing search factor within a historical memory. A fresh search factor was then generated by directly sampling within the parameter space near one of these stored values. Furthermore, an approach of retaining historically superior individuals by storing them in a historical optimal solution collection over several generations was adopted, enhancing the search and detection capabilities. The use of a multi-objective fitness function, integrating the K2-Score (Bayesian simplified score) [21] and the likelihood ratio (LR) [22] complementarity, amplified the algorithm’s capability to identify various disease models, thereby bolstering its optimization prowess.

**Figure 1:**
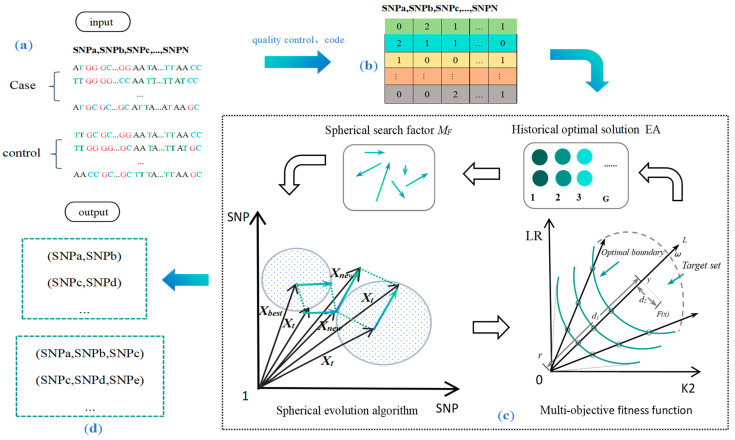
Flowchart of the SEMO algorithm for disease multi-locus SNP detection. Figure 1 shows that (**a**) is the foraging behavior of biological ants, (**b**) is the matrix of ground SNP data after quality control coding, (**c**) is the flowchart of the spherical evolutionary multi-objective algorithm, and (**d**) is the experimental results of disease multi-locus SNP interactions detected with SEMO.

The SEMO algorithm workflow is shown in Figure 1.

### 2.1. Problem Definition

Multi-locus SNP interactions are defined as phenotypic effects of nonlinear interactions of multiple SNPs. Identifying SNP interactions and revealing their corresponding genes allow further exploration of the protein functions regulated by these genes and their genetic effects and is one of the important ways to understand the pathogenesis of complex diseases. For multi-locus SNP interaction analyses, our goal was to identify the most significant set of combinations of multiple SNPs (epistatic interactions) associated with a phenotype among all SNP combinations.

GWAS use genotypic data that encode the genetic information about each individual, as well as phenotypic data that measure the quantitative characteristics of the individual. The genotypic data of interest in this paper were case–control studies of double alleles. In the raw data, A, B were used for the primary allele and a, b for the secondary allele. The genotypes of the samples were coded as 0, 1, and 2 based on the number of minor alleles at each locus. The multi-locus SNP interaction data problem can be represented in a matrix as:

(1) $$D=\left(X_{i,j},Y_{i}\right)=\left[\begin{array}{c} X_{1,1},X_{1,2}\ldots \ldots \ldots \ldots \ldots X_{1,j},Y_{1}\\ X_{2,1},X_{2,2}\ldots \ldots \ldots \ldots \ldots X_{2,j},Y_{2}\\ \ldots \ldots \ldots \ldots \ldots \ldots \ldots \ldots \ldots \ldots \ldots \ldots \\ X_{i-1,1},X_{i-1,2}\ldots \ldots X_{i-1,j},Y_{i-1}\\ X_{i,1},X_{i,2}\ldots \ldots \ldots \ldots \ldots \ldots X_{i,j},Y_{i} \end{array}\right]$$

where i denotes the number of samples and j denotes the number of SNP markers. $X_{i,j}$ ∈ (0, 1, 2), the pure primary allele, is denoted as 0; the heterozygous allele is denoted as 1; and the pure secondary allele is denoted as 2. $X_{i,j}$ is the genotype of the *j*-th SNP and the *i*-th sample in dataset D. The phenotypic variable$\;Y_{i}\;$is used to denote the disease status of sample i corresponding to its SNP, where $Y_{i}$ ∈ (0, 1). Cases are denoted as 1, and controls are denoted as 0.

### 2.2. Spherical Evolution Search Style

The spherical evolutionary multi-objective (SEMO) algorithm uses a spherical global search strategy, which is an improved version of the spherical search style in the article [23], and it uses a spherical-search-based operator. Its spherical search methodology involves the continuous adjustment of the radius and angle of a circle to explore the entirety of a given region. Three vectors, denoted as $X_{r1}$, $X_{r2}$, and $X_{r3}$, were randomly selected from the overall population. Assuming $X_{r1}$ as the initial vector, a spherical region with a radius of ${\left\|X_{r2}-X_{r3}\right\|}_{2}$ was explored, yielding a novel vector, $X_{new}$, which superseded the former vector, $X_{old}$. The paradigm of the spherical evolutionary search is exemplified as follows:

(2) $${SS}_{3}(A_{i,j},B_{i,j})={ScaleFun}_{i,j}()\cdot \|A_{i,*}-B_{i,*}{\|}_{2}\cdot \prod_{k=j}^{dim-1}\sin\left(\theta_{j}\right),j=1$$

(3) $${SS}_{3}(A_{i,j},B_{i,j})={ScaleFun}_{i,j}()\cdot \|A_{i,*}-B_{i,*}{\|}_{2}\cdot \cos(\theta_{j-1})\cdot \prod_{k=j}^{dim-1}\sin\left(\theta_{j}\right),1<j\leq dim-1$$

(4) $${SS}_{3}(A_{i,j},B_{i,j})={ScaleFun}_{i,j}()\cdot \|A_{i,*}-B_{i,*}{\|}_{2}\cdot cos(\theta_{j-1}),j=dim$$

Here is the refined version of the paragraph to meet academic writing standards:

Within this context, $\|A_{i,*}-B_{i,*}{\|}_{2}$ denotes the Euclidean distance between vector $A_{i,*}$ and vector $B_{i,*}$, representing the radius of a high-dimensional sphere. The function ${ScaleFun}_{i,j}()$ signifies the capability to adjust the radius length appropriately. The dimension size can be expressed as dim and θ corresponds to the angle between vector $A_{i,*}$ and vector $B_{i,*}$.

### 2.3. Initialization

The search process starts with the creation of feasible boundaries for the solution vectors and the initial vector population of randomly generated candidate solutions for population initialization. The SEMO algorithm selects loci to detect SNP interactions according to the following formula:

(5) $$X_{i,j}=X_{min,j}+{rand}_{ij}[0,1]\left(X_{max,j}-X_{min,j}\right)$$

Here, [0, 1] represents uniformly distributed random numbers ranging between 0 and 1.

### 2.4. Mutation Strategy

The SEMO algorithm adopts a mutation strategy that is a variant of the spherical search approach. The mutation vector $T_{i,j}\;$ for an individual $X_{i\;}$ can be expressed as follows:

(6) $$T_{i,j}=X_{i,j}+{SS}_{m}\left(X_{i,j},X_{pbest,j}\right)+{SS}_{m}\left(X_{r1,j},X_{r2,j}\right)$$

In this expression, $X_{r1}$ and $X_{r2}$ are mutually exclusive individuals randomly chosen from the current population. The degree of ‘pbest’ greediness depends on the control parameter ‘p’ (where p ∈ [0, 1]). Smaller values of ‘p’ indicate a greedier behavior.

Following the application of the mutation strategy to generate the mutated vector $T_{i,j}$, a trial vector $U_{i,j}$ is randomly generated. Once all trial vectors $U_{i,j}$ for the current generation G are generated, a selection operation based on the objective function values is applied to determine whether the target vector or trial vector will survive in the next generation G + 1.

(7) $$X_{i,j}^{G+1}=\left\{\begin{array}{c} U_{i,j}^{G}\;,\;if\;u_{i,j}^{G}<X_{i,j}^{G}\;\\ X_{i,j}^{G}\;otherwise \end{array}\right.$$

### 2.5. Historical Memory

#### 2.5.1. Individual Preservation Strategy for Historical Memory

To maintain diversity, an optional historical best solution collection, denoted as EA, is used. If the target vector $X_{i,j}$ outperforms the trial vector $U_{i,j}$, it is retained in the historical best solution collection EA. When using this collection, $X_{r2,j}$ is selected from the union of the population P and the historical collection A. The size of the collection is set to twice the population size. When the size of collection A exceeds the capacity of EA, randomly selected individuals are removed to accommodate new ones.

(8) EA=PUA

#### 2.5.2. Parameter Self-Adaptive Strategy for Historical Memory

In each generation, the search factor $F_{i}$ values for successfully generated trial vectors in that generation are recorded in a set. Upon the generation’s completion, $m_{F}$ is updated as follows:

(9) $$m_{F}=\frac{\sum_{k=1}^{\left|S\right|}{⍵}_{k}.S_{k}^{2}}{\sum_{k=1}^{\left|S\right|}{⍵}_{k}.S_{k}}$$

(10) $${⍵}_{k}=\frac{∆f_{k}}{\sum_{k=1}^{\left|S\right|}∆f_{k}}$$

(11) $$∆f_{k}=\left|f\left(u_{k}\right)-\right.\left.f\left(x_{k}\right)\right|$$

Here, $S_{K}$ represents the number of winning individuals in the current population, ${⍵}_{k}$ represents the weight of winning individuals, and the fitness function value f represents the fitness function value of winning individuals. $∆f_{k}$ is the incremental fitness value of the winning individual, $x_{k}$ refers to the winning individual of the target vector in generation g, and $u_{k}$ is the winning individual of the trial vector in generation G + 1.

At the start of the search, $m_{F}$, equipped with H historical memories, is initialized to 0.5. Throughout the search process, the historical memory set $M_{F}$ undergoes the following adjustments:

(12) $$M_{F}=\{m_{F1}{,m}_{F2}{,\cdots ,m}_{Fn}\}$$

Index k (1 ≤ k ≤ H) determines the position of the historical memory parameter set to be updated, where H represents the number of historical memories $m_{F}$. At the start of the search, k is initialized to 1. Whenever a new element is inserted into the historical records, k is incremented. If k > H, it is set back to 1. In generation G, the *i*-th element of the parameter set within historical memory is updated. During $m_{F}$ updates, if in generation G, no individual can generate trial vectors superior to their parents, i.e., S = ∅; the parameter set within historical memory remains unaltered; and this position learns from the previous position’s value.

In each generation, the control parameter $F_{i}$ used by each individual $X_{i}$ is first randomly selected from the range [1, H], for which the following formula is applied for generation:

(13) $$F_{i}={randc}_{i}(m_{F},0.1)$$

Here, $F_{i}$ is a random number generated from the Cauchy distribution. If $F_{i}$ > 1, it is set to 1. If $F_{i}$ ≤ 0, it is regenerated until a valid value is achieved. Here, 0.1 represents the scaling parameter. $m_{F}$ is randomly selected from the historical memory set $M_{F}$.

### 2.6. K2-Score

The Bayesian network model is a lightweight computational method for evaluating the association between SNP combinations and disease states with high discriminative accuracy [17]. Cooper proposed the K2 algorithm [21], which applies Bayesian scoring and a hill-climbing search to optimize the network model, where the scoring function is known as the K2-Score. In this study, the K2-Score based on Bayesian network was expressed as the following equation:

(14) $$K2-Score=\prod_{i=1}^{I}\frac{\left(J-1\right)!}{{(N}_{i}+J-1)!}\prod_{j=1}^{J}N_{ij}!$$

where I is the number of all genotype combinations of SNPs and J is the phenotypic variable indicating the number of disease states. GWAS data usually contain only samples in diseased and control states, so J is usually 2; $N_{i}$ is the number of observed combinations of SNPs in the *i*-th genotype.$N_{ij}$ is the number of *i*-th genotype SNP combinations observed for the *j*-th disease-state-associated phenotype.

The lower the K2-Score value, the higher the association between SNP combinations and disease states.

### 2.7. Likelihood Ratio (LR-Score) 

The likelihood ratio (LR) is a well-established statistical test for checking whether the parameters reflect the true constraints. As shown by Agresti [22] in the Categorical Data Analysis book, the essence of the likelihood ratio is to compare the maximum value of a likelihood function with constraints to the maximum value of a likelihood function without constraints. Specifically, it describes the ratio of observed data to expected data in a particular test problem [24].

The LR score was used as a composite metric for identifying SNP interactions with superordinate effects. It was used to statistically compare the maximum likelihood difference between unrestricted and restricted models [20,24]. In the setup of this paper, the unconstrained model consisted of the frequencies observed in the data and the constrained model consisted of the frequencies expected under the original assumption of no association. The LR was calculated [20] as follows:

(15) $$LR=2\sum_{i}^{I}\sum_{j}^{J}N_{ij}\ln\left(\frac{N_{ij}}{E_{ij}}\right)$$

where $N_{ij}$ and $E_{ij}$ denote the number of genotypes observed and the expected number of genotypes, respectively, when the SNP combination presents the *i*-th genotype and the phenotype presents the *j*-th state. $E_{ij}$ can be obtained according to the Hardy–Weinberg principle. An example of the column linkage table for the SNP combination model is shown in Supplementary Data Table S1.

The lower the LR statistic, the stronger the degree of association between the SNP combination and the phenotype.

### 2.8. Multi-Objective Fitness Function

Due to the diversity of disease models, single-objective methods may have potential disease model preference problems when they are used for topicality detection. In this study, a multi-objective fitness function based on the decomposition idea was adopted and combined two functions (K2-Score and LR-Score) as the objective function for the evolutionary iteration of the algorithm, and individuals with lowest K2 and LR-Score values were retained during the evolutionary process. The K2-Score and LR-Score functions are interactive, and their combination facilitates improved discriminatory performance for combinations of pathogenic SNPs with complementary mechanisms [20].

The multi-objective fitness function based on the decomposition idea was proposed by Qing fu Zhang [25] in 2007. The main idea is to decompose a multi-objective optimization problem into several scalar optimization subproblems and optimize them simultaneously, where each sub-problem is optimized using only the information about several adjacent sub-problems. In this study, the multi-objective optimization problem was described as:

(16) $$minimizeF\left(X\right)={\left(K2,LR\right)}^{K}$$

where *K* refers to the number of weighting vectors in the neighborhood of each weighting vector.

The decomposition-based multi-objective problem can be described as:

(17) $$minimizeD\left(x\left|\omega ,r\right.\right)=d_{1}+\theta d_{2}x∊\Omega$$

(18) $$d_{1}=\frac{\left\|{\left(r-F\left(X\right)\right)}^{k}⍵)\right\|}{\left\|⍵\right\|}$$

(19) $$d_{2}=\left\|F\left(X\right)-(r-d_{1}⍵)\right\|$$

where Ω denotes the decision space, ⍵ denotes the weight vector, r is the reference point, and θ > 0 is a preset penalty parameter. Let y be the projection of $F\left(X\right)$ on the line L, $d_{1}$ be the distance between r and y, and $d_{2}$ be the distance between $F\left(X\right)$ and L. $F\left(X\right)$ is the objective function that combines the K2-Score and the LR-Score as the evolutionary iteration of the algorithm. As represented in Figure 1c, $F\left(X\right)$ serves as the Pareto-optimal objective vector, and our goal was to push $F\left(X\right)$ as high as possible to the boundary of the achievable objective set.

## 3. Results and Discussion

The performance of the SEMO algorithm was compared with six other state-of-the-art SNP interaction detection algorithms (i.e., EACO [19], EpiACO [17], FDHEIW [15], MP-HS-DHSI [20], NHSA-DHSC [18], and SNPHarvester [10]) on simulated datasets with different disease models and experimentally validated on a breast cancer dataset from the real Wellcome Trust Case Control Consortium (WTCCC).

### 3.1. Assessment Metrics

To assess the ability of various methods of detecting epistasis, power was used as one of the assessment metrics. Power is a measure of the ability to detect combinations of disease-causing SNPs from genomic data, denoted as:

(20) $$Power=\frac{\#S}{\#T}$$

where #S is the number of pathogenic SNP combinations detected from #T datasets. Each dataset includes one pathogenic SNP combination. #T denotes the number of datasets generated from the same model parameters (#T is set to 100), power1 is the detection accuracy of each algorithm, power2 is the detection accuracy validated with the G-test on the basis of each algorithm, and power3 is the detection accuracy validated with MDR [26] on the basis of each algorithm.

To avoid the one-sidedness of a single evaluation metric, other indexes, such as sensitivity (true positive rate, TPR), positive predictive value (PPV), false discovery rate (FDR), and accuracy (ACC), were used to evaluate performance. The assessment metrics are defined in the following equations:

(21) $$TPR=\frac{TP}{TP+FN}$$

(22) $$PPV=\frac{TP}{TP+FP}$$

(23) $$ACC=\frac{TP+TN}{TP+TN+FN+FP}$$

(24) $$FDR=\frac{FP}{TP+FP}$$

(25) $$F1=\frac{2}{1/TPR+1/PPV}$$

where TP is the number of correctly recognized disease model SNP combinations, TN is the number of correctly recognized non-disease model SNP combinations, FP is the number of incorrectly recognized non-disease model SNP combinations, and FN is the number of incorrectly recognized disease model SNP combinations.F1 combines the two indexes of TPR and PPV, and when F1 is higher, it indicates that the method is more effective.

### 3.2. Experimental on Simulated Data

To evaluate the SEMO algorithm, 22 different types of disease model simulation data were used in the simulation experiments. These contained 12 disease models with marginal effects (DME) and 10 disease models without marginal effects (DNME). SEMO was evaluated against six algorithms (EACO [19], EpiACO [17], FDHEIW [15], MP-HS-DHSI [20], NHSA-DHSC [18], and SNPHarvester [10]) on simulated datasets with varying heritability (h^2^) and minor allele frequency (MAF) using metrics such as power, the TPR, the ACC, the FDR, the F1-score, and run time.

By setting different heritability (h^2^) and minor allele frequency (MAF) values, we randomly generated 100 different simulated datasets using GAMETES_2.1 [27] software, which generates datasets containing specific two-locus SSIs with random architectures. The sample size of the simulated dataset was 1600, which contained 800 controls and 800 cases. The SNP number for each sample was equal to 1000. Depending on the disease model setup, each dataset included a pair of interacting SNP combinations (M0P0 and M1P1), and the SNPs were generated based on a uniformly selected MAF in (0.01, 0.5).

#### 3.2.1. Disease Models without Marginal Effects (DNME)

DNME indicate that individual SNPs have no main effect but that several specific SNPs have a strong upward effect when combined together [28,29]. In the DNME, we generated 10 simulated datasets with MAFs set to 0.2 and 0.4 for disease-relevant loci and 0.01, 0.05, 0.2, and 0.4 for heritability h^2^. The MAFs for disease-unrelevant loci also obeyed the uniform distribution of [0.01, 0.5]. The exogeneity values of the DNME for the nine different parameters are shown in Supplementary Data Table S2.

#### 3.2.2. Disease Models without Marginal Effects (DME)

DME usually refer to models in which one or more SNPs have marginal effects but the interaction effect is stronger for all SNPs combined. In the DME, we set the MAFs of disease-associated loci to 0.05, 0.1, 0.2, and 0.5 to generate different simulated datasets, while the MAFs of disease-unassociated loci obeyed a uniform distribution of [0.01, 0.5]. The minor allele frequency (MAF) is the frequency of occurrence of a minor common allele in a given population. Prevalence is the proportion of a given population found to be affected by a disease. Prevalence P(D) is the probability that a specific population is affected by an SNP-interacting disease model. Heritability h^2^ is the phenotypic change affected by the SNP-interacting disease model. The different parameter settings for the 12 DME are in Supplementary Data Table S3.

#### 3.2.3. Analysis of Performance Indicators for Simulation Experiments

**Figure 2:**
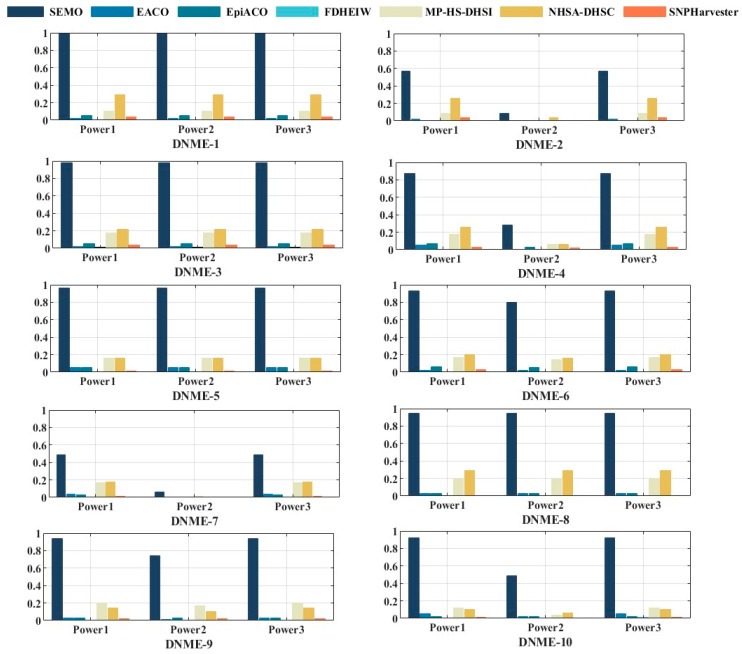
The power comparison of SEMO with six algorithms in DNME.

The experimental results showed that for the DNME, the SEMO algorithm had the highest detection ability in the 10 disease models without marginal effects, which was much higher than the other six algorithms, as can be seen in Figure 2. This is attributed to the fact that our algorithm has been debugged with multiple parameters and the dynamic allocation mechanism allows the algorithm to adaptively choose the appropriate search operation according to the characteristics of the model, resulting in better test performance compared to other algorithms. This may also be related to the property of DNME of having no marginal effects. The SEMO algorithm’s ability to detect disease-causing SNP combinations from genomic data is improved compared to the other algorithms.

Table 1 shows that the SEMO algorithm outperformed other comparison methods, not only in terms of detection accuracy, but also in terms of the TPR and PPV, resulting in an excellent performance of 75% in terms of the overall *F*1 measurement. The higher *F*1 of the SEMO algorithm compared to other algorithms indicates that the test method is more effective. According to the evaluation criteria of the FDR, SEMO outperformed the other six algorithms and had the smallest false discovery rate. The specific experimental results of the TPR, PPV, ACC, FDR, and *F*1 for the 10 DNME are shown in Supplementary Data Table S4.

**Figure 3:**
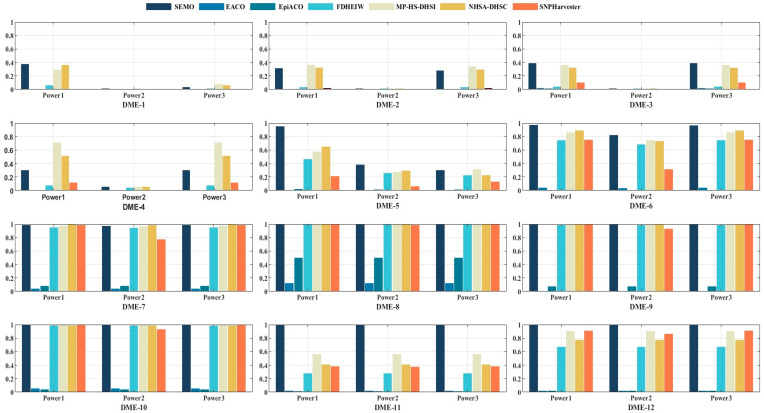
The power comparison of SEMO with six other algorithms in DME.

As shown in Figure 3, power1, power2, and power3 of the SEMO algorithm were higher than those of the other six algorithms in most of the DME, indicating that our method has better searching ability than the other six algorithms. The ability of the SEMO algorithm to detect disease-causing SNP combinations from genomic data is improved.

However, except for the DME at h^2^ = 0.005, which may be due to the fact that tiny h_2_ and MAF values may make the SEMO algorithm perform poorly, the results in Figure 3 showed that SEMO has better performance at high h2 and MAF values. Table 1 shows that SEMO’s ACC results were not ideal, but it had the best PPV as well as the smallest FDR, with marginal effects on the 12 disease models compared to the other six algorithms. This result suggests that SEMO can relatively accurately detect those SNP combinations that are indeed associated with diseases. The SEMO algorithm had an *F*1-score of 66% in the DME, outperforming most algorithms. The specific experimental results of the TPR, PPV, ACC, FDR, and *F*1 for the 12 DME are shown in Supplementary Data Table S5.

**Figure 4:**
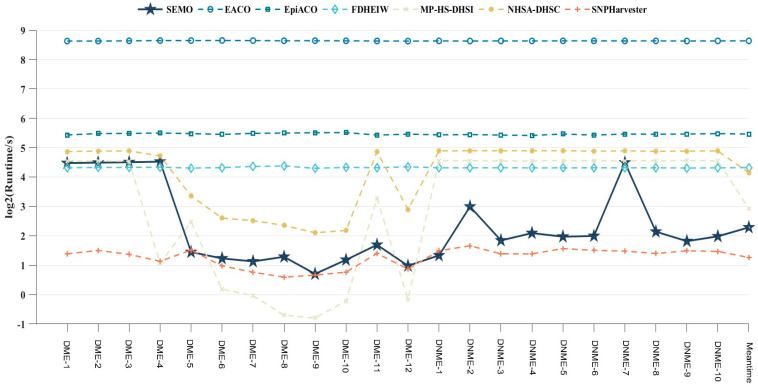
Run time and average run time of 7 algorithms in 22 disease models.

In terms of run time, the results are shown in Figure 4. Compared with the other six algorithms, the SEMO algorithm had the shortest run time in almost all disease models. The SEMO algorithm had a slightly longer run time than the MP-HS-DHSI algorithm in DME-4 and DME-6∼DME-10. However, the SEMO algorithm was far superior to the MP-HS-DHSI algorithm in terms of detection capability and other metrics, the average run time of the SEMO algorithm was faster and more stable than that of the MP-HS-DHSI algorithm, and the average run time of the SEMO algorithm was only slightly shorter than that of the SNPHarvester algorithm, but the detection performance was much better than that of the SNPHarvester algorithm. Thus, this suggests that the SEMO algorithm is more adaptable to different disease models and is somewhat faster at detecting disease-causing SNP combinations from genomic data.

In summary, most of the results demonstrate that our proposed SEMO algorithm can effectively reduce the computational burden, and its power, PPV, and FDR values are better than those of most comparative algorithms. Therefore, we believe that the SEMO algorithm may have a promising future as it can provide efficient detection performance when oriented toward the application requirements of multi-locus SNP interaction aspect detection.

### 3.3. Experiment on Real BC Data

The real dataset was derived from the breast cancer (BC) dataset from the Wellcome Trust Case Control Consortium (WTCCC) program [30]. Breast cancer is a phenomenon in which breast epithelial cells proliferate out of control under the action of various carcinogenic factors. In the advanced stage of the disease, cancer cells may undergo distant metastasis and develop into multi-organ lesions, which may directly threaten patients’ lives. Accurate identification of multi-locus SNP interactions significantly associated with BC may provide a useful reference for diagnostic and therapeutic studies of the disease. The dataset includes 15,436 SNPs from 1045 breast cancer patients and 1438 normal individuals from the 1958 birth cohort. The following quality controls were performed in this paper: among all samples, a sample was excluded if it had a genotypic deletion rate of 2%, and for an SNP, a sample was excluded if it had a genotypic deletion rate of 5% across all samples or if it had a *p*-value (Hardy–Weinberg equilibrium) < 0.0001 in the control or MAF < 0.1. After quality control, 3386 SNPs from 1045 cases and 1329 control samples from the BC dataset were used in this study.

**Figure 5:**
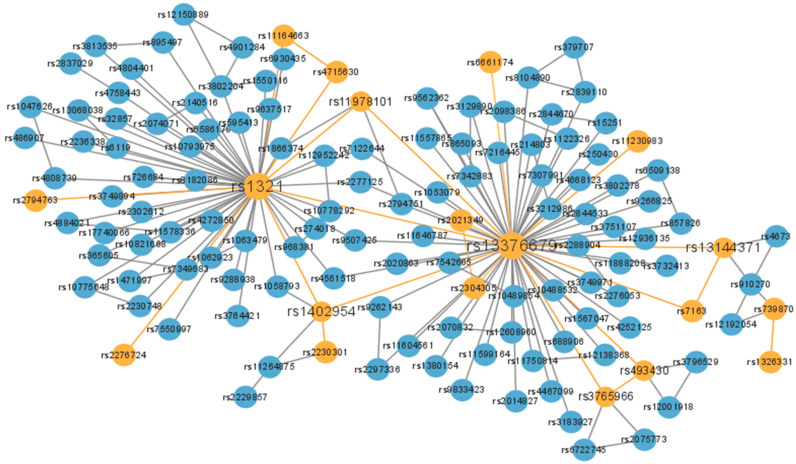
Multi-locus SNP interaction network in BC.

SNP combinatorial networks were created using Cytoscape 3.9 software http://www.cytoscape.org/ (accessed on 20 September 2023). In the SNP interaction network in Figure 5, there are 358 nodes and 368 edges. The *p*-value was determined using the Pearson chi-square test in a two-way column table to determine the significance level of multi-locus SNP interactions. The SEMO algorithm identified a number of potentially significant two- and three-locus SNP interactions from the BC dataset. Table 2 shows a representative combination of SNPs selected in this paper that are associated with BC and whose localized genes can be shown to be associated with breast cancer in this study.

Figure 5 shows that for the two-locus combination, the most frequent occurrence was rs13376679 located in the STIL gene on chromosome 1. STIL is a cilia-associated gene that can regulate tumor metastasis. In the two-site SNP combination (rs1321, rs2276724), rs1321 is located in the ALG12 gene on chromosome 22. Defects in the ALG12 gene result in mannose transferase deficiency, which can lead to a range of clinical manifestations, including growth retardation, immune deficiency, and reproductive developmental abnormalities. rs2276724 is located in the ALDH1L1 gene on chromosome 3. Loss of ALDH1L1 gene function or expression is associated with decreased apoptosis, increased cell motility, and cancer progression. In the two-locus SNP combination (rs1402954, rs2230301), rs1402954 is located in the FBXO3 gene on chromosome 11, which has been shown to be critical for breast cancer development and clinical prevention [31]. rs2230301 is located in the EPRS1 gene, which is a key regulator of breast cancer cell proliferation as well as estrogen signaling [32].

In the three-locus SNP combination (rs13376679, rs7163, rs13144371), rs13376679 is located in the STIL gene on chromosome 1, a cilia-associated gene that regulates tumor metastasis through the HIF1α–STIL–FOXM1 axis. rs13144371 is located in the IBSP gene on chromosome 4. Studies have shown that BSP gene silencing inhibits the migration, invasion, and bone metastasis of breast cancer cells [33]. In the three-locus SNP combination (rs1321, rs4715630, rs11164663), rs11164663 is located in the COL11A1 gene on chromosome 1. COL11A1 is a novel breast cancer biomarker [34]. The absence of a corresponding gene for rs2021349 on chromosome 20 also appeared in our results, and the association of this SNP with breast cancer in combination with other SNPs has not yet been reported, which may indicate that our approach has identified new combinations of SNPs associated with breast cancer.

## 4. Conclusions

Identifying disease multi-locus SNP interactions and revealing their corresponding genes so as to further investigate the protein functions regulated by the corresponding genes and the genetic effects they denote are an important way to explore the pathogenesis of complex diseases. Therefore, proving the accuracy of detection algorithms and reducing the time complexity of detection algorithms when mining SNP interactions in large-scale data are of great significance to the problem of the combinatorial explosion of motifs. In this paper, a spherical evolutionary multi-objective algorithm for detecting disease multi-locus SNP interactions was proposed, which can effectively identify disease high-order multi-locus SNPs. Historical memory sets during the search process were stored through the search factor adaptive mechanism. A multi-objective fitness function combined with two approximate normalization methods was used to evaluate the association using K2-Score and LR-Score statistical mathematical models as the objective function for the evolutionary iteration of the algorithm, which improved the optimization ability of the algorithm. Finally, the algorithm was compared with six state-of-the-art algorithms in simulation experiments. The experimental results showed that the SEMO algorithm is able to detect SNP interactions efficiently with the shortest average run time compared to other classical algorithms, which will provide a new way to detect multi-locus SNP interactions accurately and rapidly.

In addition, the SEMO algorithm was applied to a real dataset of breast cancer (BC) and significant two-locus and three-locus SNP interactions were detected, which confirmed the feasibility of the SEMO algorithm in identifying multi-locus SNP interactions from disease data. SNP combinations whose association with breast cancer is currently unreported were also identified. However, there is still room for the SEMO algorithm to improve the speed of the search and detection of multi-disease models. In this paper, we investigated potential genetic interactions in the public data on breast cancer, attributed to limitations in the clinical information of the data that do not allow for deep grouping studies based on tumor characteristics. In future studies, we will obtain real data with more complete clinical information to identify unique or shared clinical features that can be associated with combinations of SNPs that can be genetically linked. We intend to find more powerful modeling methods and corresponding scoring functions or appropriate effective optimization strategies. These strategies can be flexibly embedded into our algorithm, which in turn will enhance the detection of different disease SNP interaction models.

## Figures and Tables

**Table 1:** Mean and standard deviation of algorithmic evaluation indicators.

Model	Algorithm	TPR		PPV		ACC		FDR		F1	
		E_-mean_	E_-sd_	E_-mean_	E_-sd_	E_-mean_	E_-sd_	E_-mean_	E_-sd_	E_-mean_	E_-sd_
DME 1–12	SEMO	0.64	0.43	0.97	0.8	0.83	0.19	0.3	0.8	0.66	0.41
	EACO	0.48	0.48	0.17	0.28	0.81	0.27	0.83	0.28	0.21	0.29
	EpiACO	0.54	0.48	0.28	0.36	0.88	0.17	0.72	0.36	30.1	0.32
	FDHEIW	0.73	0.34	0.71	0.38	0.94	0.6	0.28	0.38	0.71	0.36
	MP-HS-DHSI	0.63	0.43	0.79	0.32	0.81	0.21	0.21	0.32	0.65	0.42
	NHSA-DHSC	0.63	0.42	0.83	0.33	0.83	0.17	0.17	0.33	0.67	0.40
	SNPHarvester	0.52	0.43	0.67	0.47	0.89	0.12	0.33	0.47	0.57	0.43
DNME 1–10	SEMO	0.68	0.34	1.00	0.5	0.77	0.22	0.4	0.5	0.75	0.30
	EACO	0.57	0.45	0.70	0.46	0.98	0.02	0.30	0.46	0.61	0.43
	EpiACO	0.73	0.40	0.78	0.39	0.99	0.01	0.22	0.39	0.74	0.39
	FDHEIW	0.10	0.30	0.03	0.10	0.98	0.01	0.97	0.10	0.05	0.15
	MP-HS-DHSI	0.64	0.39	0.77	0.27	0.93	0.05	0.27	0.27	0.65	0.34
	NHSA-DHSC	0.65	0.37	0.84	0.29	0.92	0.08	0.16	0.29	0.69	0.34
	SNPHarvester	0.47	0.48	0.50	0.50	0.99	0.01	0.50	0.50	0.48	0.48

E_-mean_, mean value of evaluation indicators; E_-sd_, standard deviation of evaluation indicators.

**Table 2:** Multi-locus SNP interactions in BC represent combinations.

SNPs Interactions	Chromosome	Related Genes	*p*-Value
(rs13376679, rs2242637)	(1, 1)	(STIL, ARTN)	<1 × 10^−100^
(rs1402954, rs2230301)	(11, 1)	(FBXO3, EPRS1)	<1 × 10^−100^
(rs1321, rs2276724)	(2, 3)	(ALG12, ALDH1L1)	<1 × 10^−100^
(rs13376679, rs661174)	(1, 18)	(STIL, N/A)	<1 × 10^−100^
(rs13376679, rs7163, rs13144371)	(1, 1, 4)	(STIL, EBNA1BP2, IBSP)	<1 × 10^−100^
(rs1321, rs4715630, rs11164663)	(22, 6, 1)	(ALG12, DST, COL11A1)	<1 × 10^−100^
(rs1321, rs11978101, rs13376679)	(22, 6, 1)	(ALG12, SDK1, STIL)	<1 × 10^−100^
(rs13376679, rs2290501, rs7038903)	(1, 1, 9)	(STIL, HSPG2, SVEP1)	<1 × 10^−100^
(rs13376679, rs1321, rs1402954)	(1, 22, 11)	(STIL, ALG12, FBXO3)	<1 × 10^−100^
(rs13376679, rs2304305, rs2021349)	(1, 1, 20)	(STIL, ACOT11, N/A)	<1 × 10^−100^

N/A indicates that the related gene is unknown. The *p*-values were estimated using the Pearson chi-square test.

## Data Availability

The genome-wide breast cancer dataset was requested from the Wellcome Trust Case Control Consortium (WTCCC), and its accession number was “EGAD00000000013”. WTCCC datasets cannot be shared without permission from the WTCCC. The researchers interested in WTCCC datasets can also apply for them from the WTCCC https://www.wtccc.org.uk/ (accessed on 15 May 2022).

## Supplementary Materials

The following supporting information can be downloaded at https://www.mdpi.com/article/10.3390/genes15010011/s1: Table S1. Example of a contingency table of counts for an SNP model; Table S2. Parameter settings for the 10 DNME; Table S3. Parameter settings for the 12 DME; Table S4. TPR, PPV, ACC, FDR, and *F*1 values for the 10 DNME; Table S5. TPR, PPV, ACC, FDR, and F1 values for the DME.

## References

[1] Shang J., Cai X., Zhang T., Sun Y., Zhang Y., Liu J., Guan B. (2022). EpiReSIM: A Resampling Method of Epistatic Model without Marginal Effects Using Under-Determined System of Equations. Genes.

[2] Bateson W., Mendel G. (2009). Mendel’s Principles of Heredity: A Defence, with a Translation of Mendel’s Original Papers on Hybridisation.

[3] Uppu S., Krishna A., Gopalan R.P. (2018). A Review on Methods for Detecting SNP Interactions in High-Dimensional Genomic Data. IEEE/ACM Trans. Comput. Biol. Bioinform..

[4] Wang H., Wu X. (2023). IPP: An Intelligent Privacy-Preserving Scheme for Detecting Interactions in Genome Association Studies. IEEE/ACM Trans. Comput. Biol. Bioinform..

[5] Guan B., Zhao Y. (2019). Self-Adjusting Ant Colony Optimization Based on Information Entropy for Detecting Epistatic Interactions. Genes.

[6] Becker T., Schumacher J., Cichon S., Baur M.P., Knapp M. (2005). Haplotype interaction analysis of unlinked regions. Genet. Epidemiol..

[7] Milne R.L., Herranz J., Michailidou K. (2014). A large-scale assessment of two-way SNP interactions in breast cancer susceptibility using 46,450 cases and 42,461 controls from the breast cancer association consortium. Hum. Mol. Genet..

[8] Zubenko G.S., Hughes H.B., Zubenko W.N. (2010). D10S1423 identifies a susceptibility locus for Alzheimer’s disease (*AD7*) in a prospective, longitudinal, double-blind study of asymptomatic individuals: Results at 14 years. Am. J. Med. Genet. Part B Neuropsychiatr. Genet. Off. Publ. Int. Soc. Psychiatr. Genet..

[9] Cordell H.J. (2002). Epistasis: What it means, what it doesn’t mean, and statistical methods to detect it in humans. Hum. Mol. Genet..

[10] Yang C., He Z., Wan X., Yang Q., Xue H., Yu W. (2009). SNPHarvester: A filtering-based approach for detecting epistatic interactions in genome-wide association studies. Bioinformatics.

[11] Shang J., Zhang J., Lei X., Zhang Y., Chen B. (2012). Incorporating heuristic information into ant colony optimization for epistasis detection. Genes Genom..

[12] Ritchie M.D., Hahn L.W., Roodi N., Bailey L.R., Dupont W.D., Parl F.F., Moore J.H. (2001). Multifactor-dimensionality reduction reveals high-order interactions among estrogen-metabolism genes in sporadic breast cancer. Am. J. Hum. Genet..

[13] Yang C.H., Lin Y.D., Chuang L.Y. (2020). Class Balanced Multifactor Dimensionality Reduction to Detect Gene-Gene Interactions. IEEE/ACM Trans. Comput. Biol. Bioinform..

[14] Ponte-Fernandez C., Gonzalez-Dominguez J., Carvajal-Rodriguez A., Martin M.J. (2022). Evaluation of Existing Methods for High-Order Epistasis Detection. IEEE/ACM Trans. Comput. Biol. Bioinform..

[15] Tuo S. (2018). FDHE-IW: A Fast Approach for Detecting High-Order Epistasis in Genome-Wide Case-Control Studies. Genes.

[16] Wang X., Cao X., Feng Y., Guo M., Yu G., Wang J. (2022). ELSSI: Parallel SNP-SNP interactions detection by ensemble multi-type detectors. Brief. Bioinform..

[17] Sun Y., Shang J., Liu J.X., Li S., Zheng C.H. (2017). epiACO—A method for identifying epistasis based on ant Colony optimization algorithm. BioData Min..

[18] Tuo S., Zhang J., Yuan X., He Z., Liu Y., Liu Z. (2017). Niche harmony search algorithm for detecting complex disease associated high-order SNP combinations. Sci. Rep..

[19] Sun Y., Wang X., Shang J., Liu J.X., Zheng C.H., Lei X. (2020). Introducing Heuristic Information Into Ant Colony Optimization Algorithm for Identifying Epistasis. IEEE/ACM Trans. Comput. Biol. Bioinform..

[20] Tuo S., Liu H., Chen H. (2020). Multipopulation harmony search algorithm for the detection of high-order SNP interactions. Bioinformatics.

[21] Cooper G.F., Herskovits E. (1992). A Bayesian method for the induction of probabilistic networks from data. Mach. Learn..

[22] Agresti A. (2002). Introduction: Distributions and Inference for Categorical Data. Categorial Data Analysis.

[23] Tang D. (2019). Spherical evolution for solving continuous optimization problems. Appl. Soft Comput..

[24] Bush W.S., Edwards T.L., Dudek S.M., McKinney B.A., Ritchie M.D. (2008). Alternative contingency table measures improve the power and detection of multifactor dimensionality reduction. BMC Bioinform..

[25] Zhang Q., Li H. (2007). MOEA/D: A Multiobjective Evolutionary Algorithm Based on Decomposition. IEEE Trans. Evol. Comput..

[26] Abo Alchamlat S., Farnir F. (2017). KNN-MDR: A learning approach for improving interactions mapping performances in genome wide association studies. BMC Bioinform..

[27] Urbanowicz R.J., Kiralis J., Sinnott-Armstrong N.A., Heberling T., Fisher J.M., Moore J.H. (2012). GAMETES: A fast, direct algorithm for generating pure, strict, epistatic models with random architectures. BioData Min..

[28] Cordell H.J. (2009). Detecting gene-gene interactions that underlie human diseases. Nat. Rev. Genet..

[29] Culverhouse R., Suarez B.K., Lin J., Reich T. (2002). A perspective on epistasis: Limits of models displaying no main effect. Am. J. Hum. Genet..

[30] Burton P.R., Clayton D.G., Cardon L.R., Craddock N., Deloukas P. (2007). Association scan of 14,500 nonsynonymous SNPs in four diseases identifies autoimmunity variants. Nat. Genet..

[31] Niu M., He Y., Xu J., Ding L., He T., Yi Y., Fu M., Guo R., Li F., Chen H. (2021). Noncanonical TGF-β signaling leads to FBXO3-mediated degradation of ΔNp63α promoting breast cancer metastasis and poor clinical prognosis. PLoS Biol..

[32] Katsyv I., Wang M., Song W.M., Zhou X., Zhao Y., Park S., Zhu J., Zhang B., Irie H.Y. (2016). EPRS is a critical regulator of cell proliferation and estrogen signaling in ER+ breast cancer. Oncotarget.

[33] Wang J., Wang L., Xia B., Yang C., Lai H., Chen X. (2013). BSP gene silencing inhibits migration, invasion, and bone metastasis of MDA-MB-231BO human breast cancer cells. PLoS ONE.

[34] Shi W., Chen Z., Liu H., Miao C., Feng R., Wang G., Chen G., Chen Z., Fan P., Pang W. (2022). COL11A1 as an novel biomarker for breast cancer with machine learning and immunohistochemistry validation. Front. Immunol..

