# Supplementary material for: The Spherical Evolutionary Multi-Objective (SEMO) Algorithm for Identifying Disease Multi-Locus SNP Interactions
Source: Genes (Basel). 2023 Dec 20;15(1):11. doi: 10.3390/genes15010011 (PMC10815643; doi:10.3390/genes15010011)
Supplement: Supplementary file 1 [file genes-15-00011-s001.zip › genes-2704526-supplementary.pdf]

genes

Supplementary File

Supplementary File of "The Spherical Evolutionary Multi-Ob-jective (SEMO) Algorithm for Identifying Disease Multi-Locus SNP Interactions"

Fuxiang Ren [1], Shiyin Li [1], Zihao Wen [2, 3, *], Yidi Liu [1] and Deyu Tang [1,2, *]

[1] School of Medical Information Engineering, Guangdong Pharmaceutical University, Guangzhou 510006, China; renfuxiang163@163.com (F.R.); shiyyy0326@163.com (S.L.); 17803873294@163.com (Y.L.)

[2] School of Mathematics and Informatics, College of Software Engineering, South China University, Guangzhou 510642, China

[3] Faculty of Information Technology, Monash University, Melbourne, VIC 3800, Australia

* Correspondence: zihao.wen@scau.edu.cn (Z.W.); tangdeyu2023@scau.edu.cn (D.T.)

1. An example of a contingency table for a SNP mode

Table S1. Example of a contingency table of counts for a SNP model.

	Disease status	Genotype combination of SNP									
		0-0	0-1	0-2	1-0	1-1	1-2	2-0	2-1	2-2	Sum
(N_{ij})	**Number of Case samples**	12	10	3	5	8	4	5	6	2	55
	**Number of Control samples**	15	5	8	6	15	6	5	8	7	75
	Sum (case, control)	27	15	11	11	23	10	10	14	9	130
(E_{ij})	**Expected number of Cases**	11.4	6.3	4.7	4.7	9.7	4.2	4.2	5.9	3.8	55
	**Expected number of Controls**	15.6	8.7	6.3	6.3	13.3	5.8	5.8	8.1	5.2	75

2. Disease modelling without marginal effects (DNME)

The DNME disease model indicates that individual SNPs have no main effect, but several specific SNPs have a strong upward effect when combined together[28; 29]. In the DNME model, we generated 10 simulated datasets with MAFs set to 0.2 and 0.4 for disease-relevant loci and 0.01,0.05,0.2 and 0.4 for heritability h^2. The MAFs for disease irrelevant loci also obeyed the uniform distribution of [0.01, 0.5]. The exogeneity values of the DNME disease model for the nine different parameters were as shown in Table S1.

Table S2. Parameter settings for 12 DNME models

DNME-1	P(D)=0.1, h^2=0.05, MAF=0.2			DNME-2	P(D)=0.1, h^2=0.01, MAF=0.2		
	AA	Aa	aa		AA	Aa	aa
**BB**	0.6377	0.4884	0.3826	**BB**	0.2216	0.2758	0.1414
**Bb**	0.4638	0.7645	0.9566	**Bb**	0.2587	0.1690	0.4013
**bb**	0.5798	0.5624	0.7189	**bb**	0.2781	0.1279	0.4196
DNME-3	P(D)=0.1, h^2=0.05, MAF=0.2			DNME-4	P(D)=0.1, h2=0.01, MAF=0.2		
	AA	Aa	aa		AA	Aa	aa
**BB**	0.4988	0.6388	0.7649	**BB**	0.1391	0.1882	0.2214
**Bb**	0.6665	0.3887	0.0831	**Bb**	0.1901	0.1114	0.0198
**bb**	0.5430	0.5265	0.9533	**bb**	0.2056	0.0514	0.2530
DNME-5	P(D)=0.1, h^2=0.05, MAF=0.2			DNME-6	P(D)=0.1, h^2=0.01, MAF=0.4		
	AA	Aa	aa		AA	Aa	aa
**BB**	0.2121	0.3503	0.1161	**BB**	0.1032	0.0634	0.1242
**Bb**	0.3364	0.0543	0.4948	**Bb**	0.0978	0.0858	0.0693

**bb**	0.2272	0.2727	0.4948	**bb**	0.0210	0.1467	0.0595
DNME-7	**P(D)=0.1, h^2=0.01,MAF=0.4**			DNME-8	**P(D)=0.1, h^2=0.01, MAF=0.4**		
	**AA**	**Aa**	**aa**		**AA**	**Aa**	**aa**
**BB**	0.1852	0.2908	0.2340	**BB**	0.0731	0.0418	0.0146
**Bb**	0.2860	0.2009	0.2770	**Bb**	0.0240	0.0639	0.0591
**bb**	0.2486	0.2661	0.1657	**bb**	0.0682	0.0188	0.0946
DMNE-9	**P(D)=0.1, h^2=0.01, MAF=0.4**			DNME-10	**P(D)=0.1, h^2=0.01, MAF=0.4**		
	**AA**	**Aa**	**aa**		**AA**	**Aa**	**aa**
**BB**	0.0462	0.1275	0.0694	**BB**	0.0950	0.1222	0.1267
**Bb**	0.1150	0.0667	0.0971	**Bb**	0.0973	0.1294	0.0999
**bb**	0.1067	0.0691	0.1085	**bb**	0.2014	0.0439	0.1222

* P(D) is prevalence, h^2 is heritability, and MAF stands for minor allele frequency.

3. Disease modelling without marginal effects (DME)

DME disease model usually refers to a model in which one or more SNPs have marginal effects, but the interaction effect is stronger for all SNPs combined. In the DME model, we set the MAFs of disease-associated loci to 0.05, 0.1, 0.2, and 0.5 to generate different simulated datasets, while the MAFs of disease-unassociated loci obeyed a uniform distribution of [0.01, 0.5]. Minor Allele Frequency (MAF) is the frequency of occurrence of a minor common allele in a given population. Prevalence is the proportion of a given population found to be affected by a disease. Prevalence P(D) is the probability that a specific population is affected by a SNP-interacting disease model. Heritability h2 is the phenotypic change affected by the SNP-interacting disease model. The different parameter settings for the 12 DME models are in Table S2.

Table S3. Parameter settings for 10 DME models

DME-1	**P(D)=0.1, h^2=0.005, MAF=0.05**			DME-2	**P(D)=0.1, h^2=0.005, MAF=0.1**		
	**AA**	**Aa**	**aa**		**AA**	**Aa**	**aa**
**BB**	0.0980	0.0980	0.0980	**BB**	0.0960	0.0960	0.0960
**Bb**	0.0980	0.2989	0.5222	**Bb**	0.0960	0.1971	0.2824
**bb**	0.0980	0.5222	0.9121	**bb**	0.0960	0.2824	0.4047
DME-3	**P(D)=0.1, h^2=0.005, MAF=0.2**			DME-4	**P(D)=0.1, h^2=0.005, MAF=0.5**		
	**AA**	**Aa**	**aa**		**AA**	**Aa**	**aa**
**BB**	0.0921	0.0921	0.0921	**BB**	0.0782	0.0782	0.0782
**Bb**	0.0921	0.1445	0.1810	**Bb**	0.0782	0.1054	0.1223
**bb**	0.0921	0.1810	0.2266	**bb**	0.0782	0.1223	0.1420
DME-5	**P(D)=0.1, h^2=0.02, MAF=0.05**			DME-6	**P(D)=0.1, h^2=0.02, MAF=0.1**		
	**AA**	**Aa**	**aa**		**AA**	**Aa**	**aa**
**BB**	0.0958	0.0958	0.0958	**BB**	0.0918	0.0918	0.0918
**Bb**	0.0958	0.5331	0.5331	**Bb**	0.0918	0.3192	0.3192
**bb**	0.0958	0.5331	0.5331	**bb**	0.0918	0.3192	0.3192
DME-7	**P(D)=0.1, h^2=0.02, MAF=0.2**			DME-82	**P(D)=0.1, h^2=0.02, MAF=0.5**		
	**AA**	**Aa**	**aa**		**AA**	**Aa**	**aa**
**BB**	0.0804	0.1918	0.1918	**BB**	0.0519	0.0519	0.0519
**Bb**	0.1918	0.0804	0.0804	**Bb**	0.0519	0.1374	0.1374
**bb**	0.1918	0.0804	0.0804	**bb**	0.0519	0.1374	0.1374
DME-9	**P(D)=0.1, h^2=0.02, MAF=0.05**			DME-10	**P(D)=0.1, h^2=0.02, MAF=0.1**		

	**AA**	**Aa**	**aa**		**AA**	**Aa**	**aa**
**BB**	0.0804	0.1918	0.1918	**BB**	0.0717	0.1636	0.1636
**Bb**	0.1918	0.0804	0.0804	**Bb**	0.1636	0.0717	0.0717
**bb**	0.1918	0.0804	0.0804	**bb**	0.1636	0.0717	0.0717
DME-11	**P(D)=0.1, h^2=0.02, MAF=0.2**			DME-12	**P(D)=0.1, h^2=0.02, MAF=0. 5**		
	**AA**	**Aa**	**aa**		**AA**	**Aa**	**aa**
**BB**	0.0608	0.1459	0.1459	**BB**	0.0671	0.1548	0.1548
**Bb**	0.1459	0.0608	0.0608	**Bb**	0.1548	0.0671	0.0671
**bb**	0.1459	0.0608	0.0608	**bb**	0.1548	0.0671	0.0671

* P(D) is prevalence, h^2 is heritability, and MAF stands for minor allele frequency.

4. Analysis of performance indicators for simulation experiments

4.1 TPR, PPV, ACC, FDR, and F1 values for DNME disease models

The specific experimental results of TPR, PPV, ACC, FDR and F1 for 12 of these DNME models are shown in Table S3.

Table S4. TPR, PPV, ACC, FDR, and F1 values for DNME disease models

**algorithms**	**indicators**	**DNME1**	**DNME2**	**DNME3**	**DNME4**	**DNME5**	**DNME6**	**DNME7**	**DNME 8**	**DNME 9**	**DNME10**
**SEMO**	**TPR**	1.00	0.16	1.00	0.32	1.00	0.86	0.12	1.00	0.79	0.53
	**SPC**	0.00	1.00	1.00	1.00	1.00	1.00	1.00	1.00	1.00	1.00
	**PPV**	1.00	1.00	1.00	1.00	1.00	1.00	1.00	1.00	1.00	1.00
	**FDR**	1.00	0.52	1.00	0.41	1.00	0.87	0.57	1.00	0.80	0.57
	**ACC**	1.00E-12	1.11E-11	1.02E-12	3.57E-12	1.04E-12	1.25E-12	1.67E-11	1.05E-12	1.35E-12	2.04E-12
	**F1**	1.00	0.27	1.00	0.49	1.00	0.93	0.22	1.00	0.88	0.70
**EACO**	**TPR**	1.00	0.00	1.00	0.00	1.00	1.00	0.00	1.00	0.33	0.40
	**SPC**	1.00	1.00	1.00	1.00	1.00	1.00	1.00	1.00	1.00	1.00
	**PPV**	1.00	0.00	1.00	0.00	1.00	1.00	0.00	1.00	1.00	1.00
	**FDR**	1.00	0.98	1.00	0.95	1.00	1.00	0.96	1.00	0.98	0.97
	**ACC**	5.00E-11	1.00	5.00E-11	1.00	2.00E-11	5.00E-11	1.00	3.33E-11	1.00E-10	5.00E-11
	**F1**	1.00	0.00	1.00	0.00	1.00	1.00	0.00	1.00	0.50	0.57
**EpiACO**	**TPR**	1.00	0.00	1.00	0.43	1.00	0.83	0.00	1.00	1.00	1.00
	**SPC**	0.99	1.00	1.00	1.00	1.00	1.00	1.00	1.00	1.00	1.00
	**PPV**	0.83	0.00	1.00	1.00	1.00	1.00	0.00	1.00	1.00	1.00
	**FDR**	0.99	1.00	1.00	0.96	1.00	0.99	0.97	1.00	1.00	1.00
	**ACC**	0.17	1.00	2.00E-11	3.33E-11	2.00E-11	2.00E-11	1.00	3.33E-11	3.33E-11	5.00E-11
	**F1**	0.91	0.00	1.00	0.6	1.00	0.91	0.00	1.00	1.00	1.00
**FDHEIW**	**TPR**	0.00	0.00	1.00	0.00	0.00	0.00	0.00	0.00	0.00	0.00
	**SPC**	0.99	1.00	0.980	0.99	0.97	0.97	0.99	0.98	0.98	0.98
	**PPV**	0.00	0.00	0.33	0.00	0.00	0.00	0.00	0.00	0.00	0.00
	**FDR**	0.99	1.00	0.98	0.99	0.97	0.97	0.99	0.98	0.98	0.98
	**ACC**	1.00	1.00	0.67	1.00	1.00	1.00	1.00	1.00	1.00	1.00
	**F1**	0.00	0.00	0.50	0.00	0.00	0.00	0.00	0.00	0.00	0.00
**MP-HS-DHSI**	**TPR**	1.00	0.00	1.00	0.333	1.00	0.82	0.06	1.00	0.85	0.33
	**SPC**	0.97	1.00	0.98	0.99	0.98	0.99	0.99	0.96	0.98	0.98
	**PPV**	0.77	0.00	0.9	0.86	0.89	0.93	0.50	0.87	0.90	0.67
	**FDR**	0.97	0.91	0.98	0.87	0.98	0.96	0.83	0.97	0.95	0.90
	**ACC**	0.23	1.00	0.10	0.15	0.11	0.07	0.5	0.13	0.11	0.33
	**F1**	0.87	0.00	0.95	0.48	0.94	0.88	0.11	0.93	0.87	0.44
**NHSA-DHSC**	**TPR**	1.00	0.15	1.00	0.23	1.00	0.80	0.00	1.00	0.71	0.60
	**SPC**	1.00	1.00	1.00	0.99	0.98	0.98	1.00	1.00	0.99	0.99
	**PPV**	1.00	1.00	1.00	0.86	0.89	0.89	0.00	1.00	0.91	0.86
	**FDR**	1.00	0.78	1.00	0.79	0.98	0.94	0.82	1.00	0.95	0.95
	**ACC**	3.45E-12	2.50E-11	4.55E-12	0.14	0.11	0.11	1.00	3.45E-12	0.09	0.14
	**F1**	1.00	0.27	1.00	0.36	0.94	0.84	0.00	1.00	0.80	0.71
**SNPHar-vester**	**TPR**	1.00	0.00	1.00	0.67	1.00	0.00	0.00	0.00	1.00	0.00
	**SPC**	1.00	1.00	1.00	1.00	1.00	1.00	1.00	1.00	1.00	1.00
	**PPV**	1.00	0.00	1.00	1.00	1.00	0.00	0.00	0.00	1.00	0.00
	**FDR**	1.00	0.96	1.00	0.99	1.00	0.97	0.99	1.00	1.00	0.99
	**ACC**	2.50E-11	1.00	2.50E-11	5.00E-11	1.00E-10	1.00	1.00	1.00	5.00E-11	1.00
	**F1**	1.00	0.00	1.00	0.80	1.00	0.00	0.00	0.00	1.00	0.00

4.2 TPR, PPV, ACC, FDR, and F1 values for DNME disease models

The specific experimental results of TPR, PPV, ACC, FDR and F1 for 12 of these DNME models are shown in Table S4.

Table S5. TPR, PPV, ACC, FDR, and F1 values for DME disease models

algorithms	indicators	DME 1	DME 2	DME 3	DME 4	DME 5	DME 6	DME 7	DME 8	DME 9	DME 10	DME 11	DME 12
SEMO	TPR	0.03	0.03	0.03	0.17	0.40	0.85	0.99	1.00	1.00	1.00	1.00	1.00
	SPC	1.00	1.00	1.00	0.97	1.00	0.67	1.00	0.00	0.00	0.00	1.00	0.00
	PPV	1.00	1.00	1.00	0.71	1.00	0.99	1.00	0.99	1.00	1.00	1.00	1.00
	ACC	0.64	0.70	0.62	0.73	0.430	0.84	0.99	0.99	1.00	1.00	1.00	1.00
	FDR	1.00 E-10	1.00 E-10	1.00 E-10	0.29	2.63 E-12	0.01	1.03 E-12	0.01	1.00 E-12	1.00 E-12	1.01 E-12	1.00 E-12
	F1	0.05	0.06	0.05	0.27	0.57	0.91	0.99	0.99	1.00	1.00	1.00	1.00
EACO	TPR	0.00	0.00	0.00	0.00	0.00	0.75	1.00	1.00	0.00	1.00	1.00	1.00
	SPC	1.00	1.00	1.00	0.98	0.99	0.958	0.70	0.06	0.36	0.705	1.00	0.91
	PPV	0.00	0.00	0.00	0.00	0.00	0.43	0.12	0.13	0.00	0.15	1.00	0.18
	ACC	1.00	1.00	0.98	0.98	0.99	0.95	0.710	0.17	0.36	0.72	1.00	0.91
	FDR	1.00 E+00	1.00 E+00	1.00 E+00	1.00 E+00	1.00 E+00	5.71 E-01	8.79 E-01	8.74 E-01	1.00 E+00	8.48 E-01	5.00 E-11	0.82
	F1	0.00	0.00	0.00	0.00	0.00	0.55	0.22	0.22	0.00	0.26	1.00	0.31
EpiACO	TPR	0.00	0.00	0.00	0.00	0.50	0.00	1.00	1.00	1.00	1.00	1.00	1.00
	SPC	1.00	1.00	1.00	1.00	1.00	0.97	0.79	0.06	0.50	0.80	1.00	0.93
	PPV	0.00	0.00	0.00	0.00	1.00	0.00	0.30	0.52	0.13	0.17	1.00	0.22
	ACC	1.00	1.00	0.99	1.00	0.99	0.97	0.81	0.53	0.53	0.81	1.00	0.93
	FDR	1.00	1.00	1.00	1.00	1.00 E-10	1.00	0.70	0.49	0.87	0.83	1.00 E-10	0.78
	F1	0.00	0.00	0.00	0.00	0.67	0.00	0.46	0.68	0.23	0.30	1.00	0.36
FDHEIW	TPR	0.00	0.33	0.25	0.71	0.57	0.92	1.00	1.00	1.00	1.00	1.00	1.00
	SPC	0.93	0.95	0.97	0.88	1.00	0.92	0.40	0.00	0.00	1.00	0.99	0.97
	PPV	0.00	0.17	0.25	0.31	1.00	0.97	0.97	1.00	0.98	1.00	0.97	0.99
	ACC	0.87	0.93	0.94	0.87	0.8	0.92	0.97	1.00	0.98	1.00	0.99	0.99
	FDR	1.00	0.83	0.75	0.69	3.85 E-12	0.03	0.03	1.00 E-12	0.02	1.02 E-12	0.03	0.02
	F1	0.00	0.22	0.25	0.44	0.72	0.94	0.98	1.00	0.99	1.00	0.98	0.99
MP-HS-DHSI	TPR	0.03	0.03	0.03	0.09	0.47	0.86	0.99-	1.00	1.00	1.00	1.00	1.00
	SPC	0.90	0.94	0.98	0.90	1.00	1.00	0.33	0.00	1.00	1.00	0.98	0.9
	PPV	0.13	0.20	0.5	0.67	1.00	1.00	0.98	1.00	1.00	1.00	0.98	0.99
	ACC	0.65	0.61	0.64	0.32	0.7	0.88	0.97	1.00	1.00	1.00	0.99	0.99
	FDR	0.88	0.80	0.50	0.33	3.70 E-12	1.35 E-12	0.02	1.00 E-12	1.01 E-12	1.01 E-12	0.02	0.01
	F1	0.05	0.05	0.05	0.15	0.64	0.93	0.99	1.00	1.00	1.00	0.99	0.99
NHSA-DHSC	TPR	0.00	0.03	0.09	0.18	0.45	0.82	0.99	1.00	1.00	1.00	1.00	1.00
	SPC	0.97	0.96	1.00	0.94	1.00	0.91	0.00	0.00	1.00	1.00	1.00	0.91
	PPV	0.00	0.25	1.00	0.75	1.00	0.99	0.99	1.00	1.00	1.00	1.00	0.98
	ACC	0.62	0.66	0.71	0.55	0.64	0.83	0.98	1.00	1.00	1.00	1.00	0.98
	FDR	1.00	0.75	3.33 E-11	0.25	3.45 E-12	0.01	0.01	1.00 E-12	1.01 E-12	1.02 E-12	2.44 E-12	0.03
	F1	0.00	0.06	0.17	0.29	0.62	0.90	0.99	1.00	1.00	1.00	1.00	0.99
SNPHar-vester	TPR	0.00	0.00	0.00	0.00	0.29	0.41	0.80	0.98	0.95	0.94	0.97	0.95
	SPC	1.00	1.00	1.00	1.00	1.00	1.00	1.00	0.00	0.00	1.00	1.00	1.00
	PPV	0.00	0.00	0.00	0.00	1.00	1.00	1.00	1.00	1.00	1.00	1.00	1.00
	ACC	1.00	0.98	0.90	0.89	0.85	0.56	0.80	0.98	0.95	0.94	0.99	0.95
	FDR	1.00	1.00	1.00	1.00	1.67 E-11	3.23 E-12	1.28 E-12	1.02 E-12	1.05 E-12	1.08 E-12	2.70 E-12	1.16 E-12
	F1	0.00	0.00	0.00	0.00	0.44	0.59	0.89	0.99	0.97	0.97	0.99	0.97
